# Annotations

**Published:** 1901-11-09

**Authors:** 


### ANNOTATIONS.

### Does Club Practice Pay?

The question whether club practice is in itself remunerative, or whether it is to be regarded as partially charitable, lies at the bottom of a good many of the difficulties which so often arise between medical men and friendly societies. There can be no doubt that in origin the doctors' clubs had in them a charitable element, and that an essential feature in the system was that it was only meant for the poor. Indeed it seems probable that many of the evils which have grown up around the system primarily arose from this very fact and from the associated implication that it was only a third-class service that was contracted for. The friendly societies, however, scout the idea, and while accepting the benefit of the old rates, refuse to restrict the system to their poorer members, maintaining that their subscriptions are a good and sufficient payment for the services to be rendered. The question then whether contract practice pays, when conducted under present conditions and at present rates, urgently demands an answer. Dr. Arthur Larking, in an address to a branch of the British Medical Association, has lately shown that, basing his estimate on the returns of many friendly societies, the number receiving sick pay works out at such a rate that, on an average, each member would be on the sick list for between 12 and 13 days each year. Here then we have something to go upon. Evidently whatever may be the amount per member which is paid to the doctor per year, it ought to be such as would give him proper remuneration for providing medicine and attendance during an illness lasting at least twelve days, and what each club doctor has to ask himself is, Does the half-crown or the four shillings which I now receive give me a proper payment for such an attendance? If it do not, and if it be maintained that all sorts and conditions of people are to be admitted to the benefit of the club, the rates should at once be raised. The figure to be asked will vary according to the style of practice a man does, the class of patients in the club, and the general standard of the district; but in any case it must be estimated on the basis of each member's payment being sufficient to cover an attendance of at least 12 days' duration. It seems quite evident that the penny a week, or roughly four shillings a year, which is so often regarded as a maximum, is really only a relic of the old days when clubs were charities.

### Ringworm in Schools.

Dr. Phineas Abraham has recently drawn attention to the prevalence of ringworm in the schools of the metropolis. He says that the majority of the subjects who come under his notice at the Blackfriars Hospital for Skin Diseases, and at the West London Hospital, have contracted their ringworm in the board schools, and he thinks that the time has come when something ought to be done in the matter. And so do we. As he justly says, it is not fair that poor people should be forced, as they are, to send their healthy children to institutions where there is every probability of their contracting such diseases, for under the present system infected children may, and often do, attend school for weeks or months, spreading ringworm, pediculosis, etc., unchecked. The obvious remedies are that in the first place every school should be periodically inspected by a skilled medical officer; and secondly, that all cases of ringworm should be isolated from healthy children, being taught in separate rooms, or better still in distinct and special schools. Some people no doubt consider that the fact that education being free is a good set-off against its being compulsory. We think, however, that the very fact that it is compulsory throws upon the education authorities the obligation of ensuring that no greater risks of infection than are unavoidable shall be run by the children, and as everybody knows this is not done at the present time. The principle of free and compulsory education was made a battle ground among ecclesiastics and educationalists for years before it was accepted, but the discussion chiefly raged around the religious aspects of the question. It is obvious enough, however, that many economic and sanitary problems are raised by it which are as yet practically untouched. Having got the children the question is, What to do with them? The ostensible object of the law was to give them a good education; the effect has been to give them some very bad infections, of which ringworm may be taken as a rough example. It is clear that if schools can properly be charged with the spread of a coarse infection like that of ringworm they must be very hot-beds for the dissemination of the more subtle disease germs, and that if reasonable justice is to be done to healthy children the School Board has got its work cut out for it for some time to come.

### The Recrudescence of Plague.

The recrudescence of plague in Liverpool and in Glasgow, together with the failure to connect the recent cases with any fresh importation of the disease, are probably to be attributed to the fact that plague, as it affects great cities, is mostly an epidemic disease continuing during long periods among certain of the lower animals, but only showing itself occasionally and sporadically among men. The repeated appearance followed by the rapid disappearance of so eminently infective disease as plague would be absolutely inexplicable did we not know that what we see, and hear about, in the form of plague as it occurs among men is but the visible fringe of an invisible epidemic which has probably been going on all the time among the rats of these infected cities. As we have before pointed out, the mere importation even of many cases of plague by ships trading with foreign countries, followed as it naturally is by immediate isolation and other precautions, is as nothing compared with the occurrence of one or two untraceable cases in a stationary population. That such cases should crop up again and again, shows, we think, almost unmistakably that the missing link is some continuing epizootic, and with the knowledge we have on the subject it is hardly to be doubted that at the present time plague is epidemic among rats both in Liverpool and Glasgow. Could not these cities bring their rats under municipal control? With their foreign trade in danger as it now is, such an enterprise might pay them better than either trams or gasworks.

